# Temporal characteristics and associated factors of discontinuation and outcomes after percutaneous coronary intervention

**DOI:** 10.3389/fphar.2024.1355231

**Published:** 2024-04-09

**Authors:** Haiyan Xu, Wanxiang Zheng, Jiangqin Tan, Min Li

**Affiliations:** ^1^ Experimental Research Center for Medical and Psychological Science, School of Psychology, Army Military Medical University, Chongqing, China; ^2^ Department of Cardiology, Southwest Hospital, Army Military Medical University, Chongqing, China; ^3^ Team 17, Group 5, School of Basic Medicine, Army Military Medical University, Chongqing, China; ^4^ Department of Military Psychology, School of Psychology, Army Military Medical University, Chongqing, China

**Keywords:** medication adherence, discontinuation, percutaneous coronary intervention, major adverse cardiovascular events, medication adherence intervention

## Abstract

**Background:** Medication adherence in patients after percutaneous coronary intervention (PCI) is suboptimal, and discontinuation is common. Information on the temporal characteristics and associated factors of discontinuation and outcomes after PCI is insufficient to improve medication adherence interventions.

**Methods:** We conducted a single-center retrospective study of post-PCI patients by telephone survey and medical record extraction. Temporal characteristics and associated factors of discontinuation and outcomes were examined by survival curve analysis, Cox regression, or time-dependent Cox regression.

**Results:** Discontinuation and major adverse cardiovascular events (MACE) after PCI had similar temporal characteristics, with the highest incidence in the first year, followed by a decline. Temporary discontinuation was associated with pre-PCI medication nonadherence (HR 1.63; 95% CI: 1.09–2.43), lack of medication necessity (HR 2.33; 95% CI: 1.44–3.78), economic difficulties (HR 2.09; 95% CI: 1.26–3.47), routine disruption (HR 2.09; 95% CI: 1.10–3.99), and emotional distress (HR 2.76; 95% CI: 1.50–5.09). Permanent discontinuation was associated with residence in rural areas (HR 4.18; 95% CI: 1.84–9.46) or small to medium-sized cities (HR 4.21; 95% CI: 1.82–9.73), lack of medication necessity (HR 10.60; 95% CI: 6.45–17.41), and side effects (HR 3.30; 95% CI: 1.94–5.62). The MACE after PCI was associated with pre-PCI hypertension (HR 1.42; 95% CI: 1.04–1.96), two coronary stents (HR 1.42; 95% CI: 1.01–1.99) or three coronary stents (HR 1.66; 95% CI: 1.11–2.49) compared to one coronary stent up to this PCI, and temporary discontinuation (≤60 months HR 2.18; 95% CI: 1.47–3.25; >60 months HR 8.82; 95% CI: 3.65–21.28).

**Conclusion:** Discontinuation and MACE after PCI have similar temporal characteristics, temporary discontinuation and permanent discontinuation have different associated factors, and the former is associated with MACE. These findings may provide guidance for medication adherence interventions.

## Introduction

Globally, and particularly in developed countries, ischemic heart disease is the leading cause of death in people over 50 years of age (Diseases and Injuries, 2020). Percutaneous coronary intervention (PCI) is widely used to treat severe ischemic heart disease and has significantly improved outcomes (Hoole and Bambrough, 2020; Writing Committee et al., 2022). After PCI, patients need to take secondary preventive medications regularly for a long period of time, and medication adherence affects the long-term cardiovascular outcomes after PCI (Xie et al., 2019; Lee et al., 2023; Zhang et al., 2023), while it is suboptimal (Zhang et al., 2023). Although effective medication adherence interventions are significantly associated with improved outcomes (Allemann et al., 2017), current medication adherence interventions after PCI are unsatisfactory (Boyd et al., 2020; Chidester et al., 2022; Ho et al., 2022) and even produce mixed results (Ho et al., 2022).

To improve the effectiveness of medication adherence interventions, a deep understanding of the nature of medication adherence, especially its time-varying nature and associated factors, is essential (Gellad et al., 2017; Bosworth et al., 2018). According to the ABC terminology, medication adherence is a dynamic process with three phases, namely, initiation, implementation, and persistence, and survival analyses, such as K-M analysis and Cox regression, are advocated to fully embody this process (Vrijens et al., 2012). Discontinuation of medication based on patients’ own decision is a common behavior of medication nonadherence after PCI (Ofori-Asenso et al., 2018; Redfors et al., 2022), with a clear time of occurrence suitable for survival analyses. According to whether the medication is taken again, it can be divided into temporary discontinuation (also be called reinitiation) (Wawruch et al., 2023) and permanent discontinuation, reflecting the phases of implementation and persistence respectively (Vrijens et al., 2012; Kronish et al., 2021). At present, the time-varying nature of these two types of discontinuation and the factors associated with them are poorly understood. Research on the temporal characteristics of discontinuation after PCI mainly focuses on statins and antiplatelet agents (Franchi and Rollini, 2022; Yamamoto et al., 2023), with less attention paid to the overall trend of secondary preventive medications. This is a disadvantage to master its integrated characteristics. More importantly, there is still relatively little research on the factors associated with discontinuation after PCI (Harris et al., 2019; Yamamoto et al., 2023), especially for different patterns of discontinuation as well as their relationships with outcomes.

The purpose of this research is to investigate the temporal characteristics and associated factors of discontinuation and outcomes in patients with ischemic heart disease after PCI using survival analysis. It is part of a post-PCI medication adherence study and was approved for medical ethics review by the First Affiliated Hospital of the Army Medical University (Southwest Hospital) (approval number: KY2020050).

## Materials and methods

### Study design and study population

A single-center, retrospective cohort design was used to evaluate the temporal characteristics and associated factors of discontinuation and outcomes in patients after PCI. Patients with ischemic heart disease who had successfully undergone PCI at Southwest Hospital, had valid contact information, and were willing to participate in the study were enrolled as subjects. We identified the final participants for the following reasons. (1) Most existing post-PCI surveys focus on the first 3 years after PCI, and there are fewer data on the follow-up of patients beyond 3 years after PCI (Zhang et al., 2021; Zhang et al., 2023). (2) Our pre-survey showed that the success rate of surveying patients more than 10 years after PCI was very low (<20%) due to patient death, change of contact phone number, and unwillingness to cooperate. Therefore, we limited the survey population to patients 4–10 years after PCI. (3) The average annual volume of PCI performed in the Department of Cardiology at Southwest Hospital 4–10 years before the time of the present survey was approximately 1,000 cases. According to our previous survey experience, the incidence of medication nonadherence in patients with ischemic heart disease is about 40%, and the incidence of medication nonadherence in patients after PCI is even lower, especially the percentage of discontinuation. If we select a certain number of patients for investigation in each surgical year, it is not easy to grasp the final amount of valid data as a whole, nor is it conducive to obtaining sufficient data reflecting the characteristics of discontinuation and clinical outcomes at a certain period of time after PCI. Therefore, we selected 2 years of the 4–10 years after PCI and all their post-PCI patients as the study population, namely, all patients in the fourth year after PCI (surgery in 2016, n = 959) and the seventh year after PCI (surgery in 2013, n = 913).

### Setting

**FIGURE 1:**
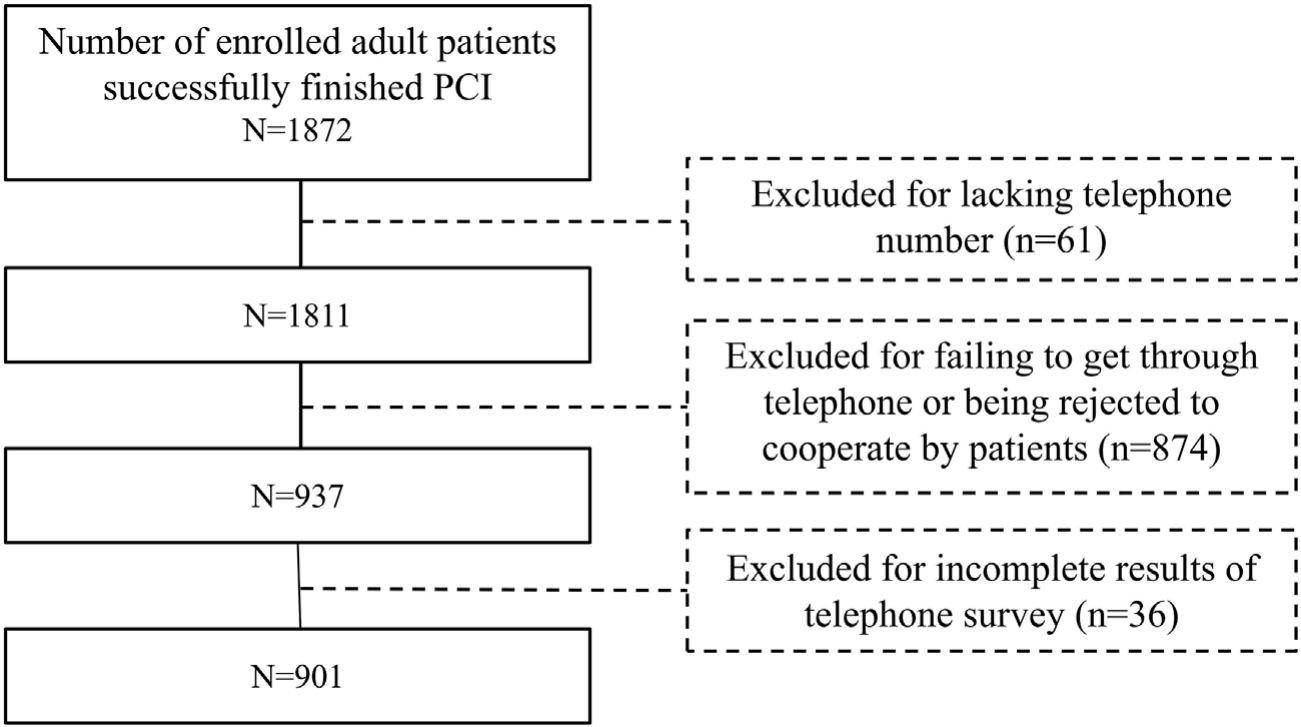
Flowchart of the study population enrollment.

According to the list of patients who successfully underwent PCI in the cardiology department of Southwest Hospital in 2013 and 2016, we found their telephone numbers from the hospital’s office computer. Patients and/or their family members were contacted by telephone, verbally solicited their opinions, and after their consent, the telephone survey was conducted and the results were recorded. Complete questionnaires from the telephone survey were extracted and completed with the medical record for baseline data at the time of PCI and information on discontinuation and outcomes after PCI. The pre-survey was conducted in May-July 2020, the centralized survey was conducted in October-December 2020, and the extraction of medical record information was centralized in January 2021, with a total of 1,811 patients surveyed. The data collection process is shown in Figure 1.

### Variables and measurements

The primary outcomes were discontinuation and MACE after PCI. Discontinuation was defined as patients stopping some or all of their prescribed medications for more than 1 week after PCI without consulting their physician or following their physician’s instructions. This includes both temporary and permanent discontinuation, with the former including resumption of medication at any time prior to our study and the latter not including resumption of medication at any time prior to our study. Due to the operability of telephone surveys, we did not differentiate between drug categories. This means that the discontinuation may include all medications prescribed by the patient’s physician for secondary prevention after PCI. MACE was defined as the composite of cardiac death, myocardial infarction, stroke, and coronary revascularization. Discontinuation and MACE after PCI were obtained by a combination of direct telephone inquiry and medical record extraction.

Related variables included baseline data at the time of PCI and barriers to medication adherence after PCI. Baseline data at the time of PCI were primarily obtained by chart review. It included demographic variables, such as sex, age, place of residence, province, education, marriage, and economic level; pre-PCI clinical characteristics, including Charlson Comorbidity Index (CCI), smoking, alcohol consumption, hypertension, hyperlipidemia, diabetes mellitus, myocardial infarction, stroke, coronary revascularization (CRV), pre-PCI medication nonadherence, meaning that the patient had any performance of these three phases of medication nonadherence before PCI; and vascular conditions up to this PCI, including number of coronary stents, noncoronary atherosclerosis or stenosis, and noncoronary stents. Barriers to medication adherence after PCI were assessed using a self-developed checklist based on the literature review, pre-survey, and interviews with clinical medical staff. The checklist includes 13 items, namely, lack of medication necessity, side effects, economic difficulties, complex prescriptions, memory decline, emotional distress, inconvenience, asymptomatic, dissatisfied with medication efficacy, disruptive routine, busy, extensive travel, and feelings of shame, each of which was self-reported as “yes” or “no” by the patient. During the telephone survey, patients were asked to rate each item according to their actual situation.

### Statistical analysis

Statistical analysis was performed using SPSS v. 22.0 (SPSS, IBM, Chicago, United States of America) and R-4.3.3 for Windows (https://posit.co/). Proportional hazards hypothesis testing and Cox regression and time-dependent Cox regression analysis of the association between MACE and potential variables were performed with R, and other statistical analyses were performed with SPSS. Categorical variables were reported as absolute values with percentages and compared by the chi-squared test. Continuous variables (age and CCI) were divided into segments according to their valid data status and treated as categorical variables. Variables with missing data were not analyzed and are not reported in the results. Kaplan-Meier survival curves (tested with log-rank and plotted with GraphPad Prism 8) were used to analyze the association between MACE after PCI and related variables, including baseline data at the time of PCI, barriers to medication adherence, and discontinuation after PCI. Variables with *p* < 0.05 in the univariate analysis were tested for the proportional hazards hypothesis and then subjected to Cox regression or time-dependent Cox regression. When the potential factors associated with discontinuation or MACE included time-dependent variables or the proportional hazards hypothesis was not met, time-dependent Cox regression model was used. Multivariable Cox regression performed with SPSS used the “Forward: LR” method to assess the association between discontinuation and related variables. The multivariable time-dependent Cox regression performed by R used the “both” direction to assess the association between MACE and related variables. A two-tailed test with *p* < 0.05 was considered as statistically significant.

## Results

A total of 901 cases with valid data were obtained in this study, 357 PCI performed in 2013 and 544 PCI performed in 2016. All stents are drug-eluting stents. The age range for PCI was 34–90 years with a median of 64 years. Post-PCI time was 48–59 months for PCI in 2016 and 84–95 months for PCI in 2013. Education, marital status, and economic level were not included in the statistical analysis due to missing data. Other baseline characteristics at the time of PCI are shown in Table 1.

**TABLE 1:** Baseline characteristics of the study cohort.

Baseline characteristics, N (%)	Total (N = 901)
*Demographics*	
Male	629 (69.8)
Age, years	
<50	106 (11.8)
50–59	202 (22.4)
60–69	339 (37.6)
≥70	254 (28.2)
Place of residence	
Large city	370 (41.1)
Small to medium-sized city	247 (27.4)
Rural area	284 (31.5)
Province	
Chongqing	703 (78.0)
Another province	198 (22.0)
*Pre-PCI clinical characteristics*	
CCI	
CCI = 0	224 (24.9)
CCI = 1	371 (41.2)
CCI = 2	197 (21.9)
CCI≥3	109 (12.1)
Smoking	509 (56.5)
Alcohol consumption	181 (20.1)
Hypertension	539 (59.8)
Hyperlipidemia	304 (33.7)
Diabetes mellitus	275 (30.5)
Myocardial infarction	257 (28.5)
Stroke	99 (11.0)
CRV	89 (9.9)
Medication nonadherence	292 (32.4)
*Vascular conditions up to this PCI*	
Number of coronary stents	
1	556 (61.7)
2	221 (24.5)
≥3	124 (13.8)
Noncoronary atherosclerosis/stenosis	282 (31.3)
Noncoronary stents	19 (2.1)

**FIGURE 2:**
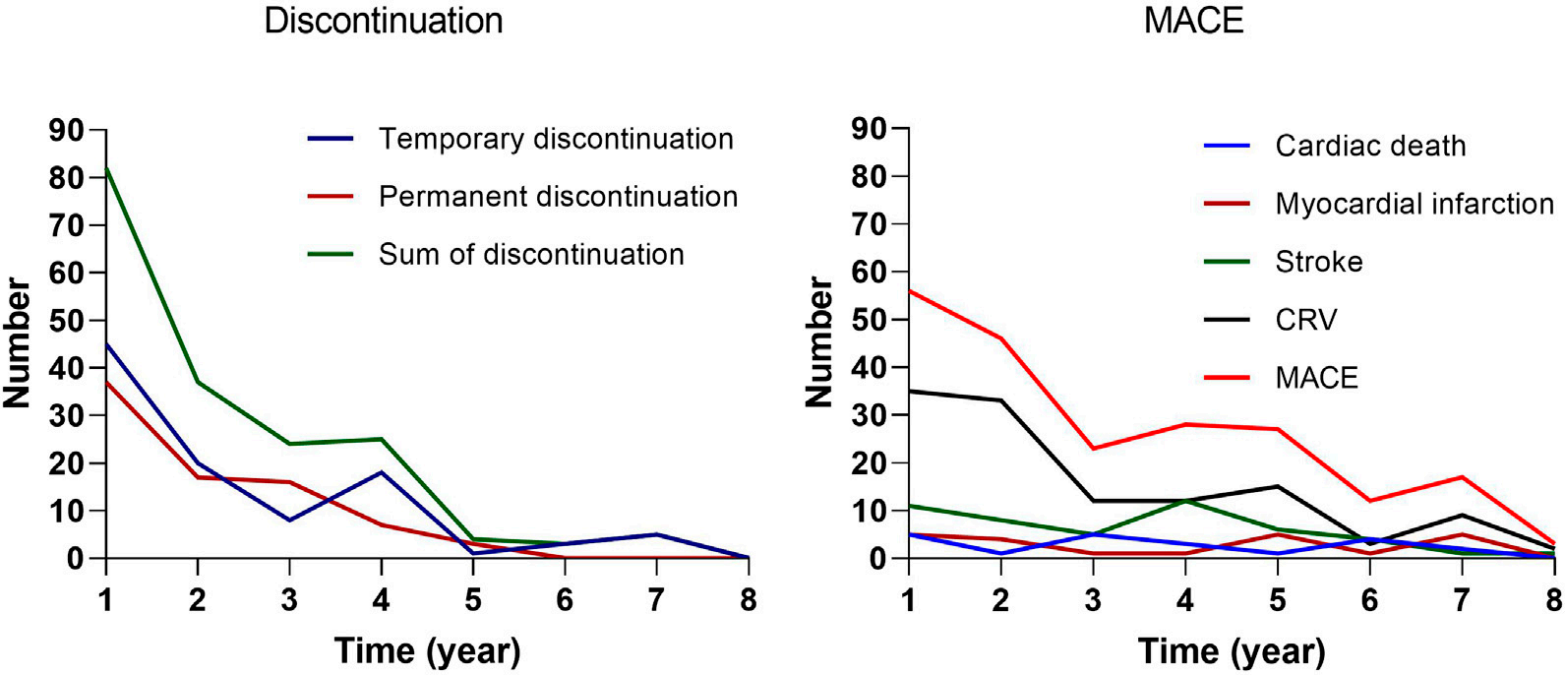
The number of discontinuation and MACE after PCI.

Number of cases and incidence of discontinuation and MACE after PCI: temporary discontinuation (100, 11.1%), permanent discontinuation (80, 8.9%), and total discontinuation (180, 20.0%); cardiac death (21, 2.3%), myocardial infarction (22, 2.4%), stroke (48, 5.3%), CRV (113, 12.5%), and total MACE (180, 20.0%). Regarding the temporal distribution (Figure 2 and Supplementary Table S1), discontinuation occurred mainly in the first year after PCI with a gradual decrease over time. The temporal distributions of cardiac death, myocardial infarction, and stroke were all roughly wavy with two to three peaks; however, their sum, CRV, and MACE, were all highest in the first year after PCI and then declined either slowly or rapidly. The common trend of discontinuation and MACE may reflect specific changes in the psychological status of patients after PCI.

Barriers to medication adherence after PCI: We removed the barriers with less valid data, and merged asymptomatic with lack of medication necessity because they overlapped a lot and the latter is the result of the former as reflected by the patients. A total of 10 barriers to medication adherence were obtained, which, in descending order of the number of valid cases and incidence rate, were lack of medication necessity (123, 13.7%), economic difficulties (75, 8.3%), memory decline (57, 6.3%), side effects (49, 5.4%), complex prescriptions (38, 4.2%), inconvenience in accessing medical care (37, 4.1%), routine disruption (33, 3.7%), emotional distress (32, 3.6%), busy (28, 3.1%), and extensive travel (24, 2.7%).

**FIGURE 3:**
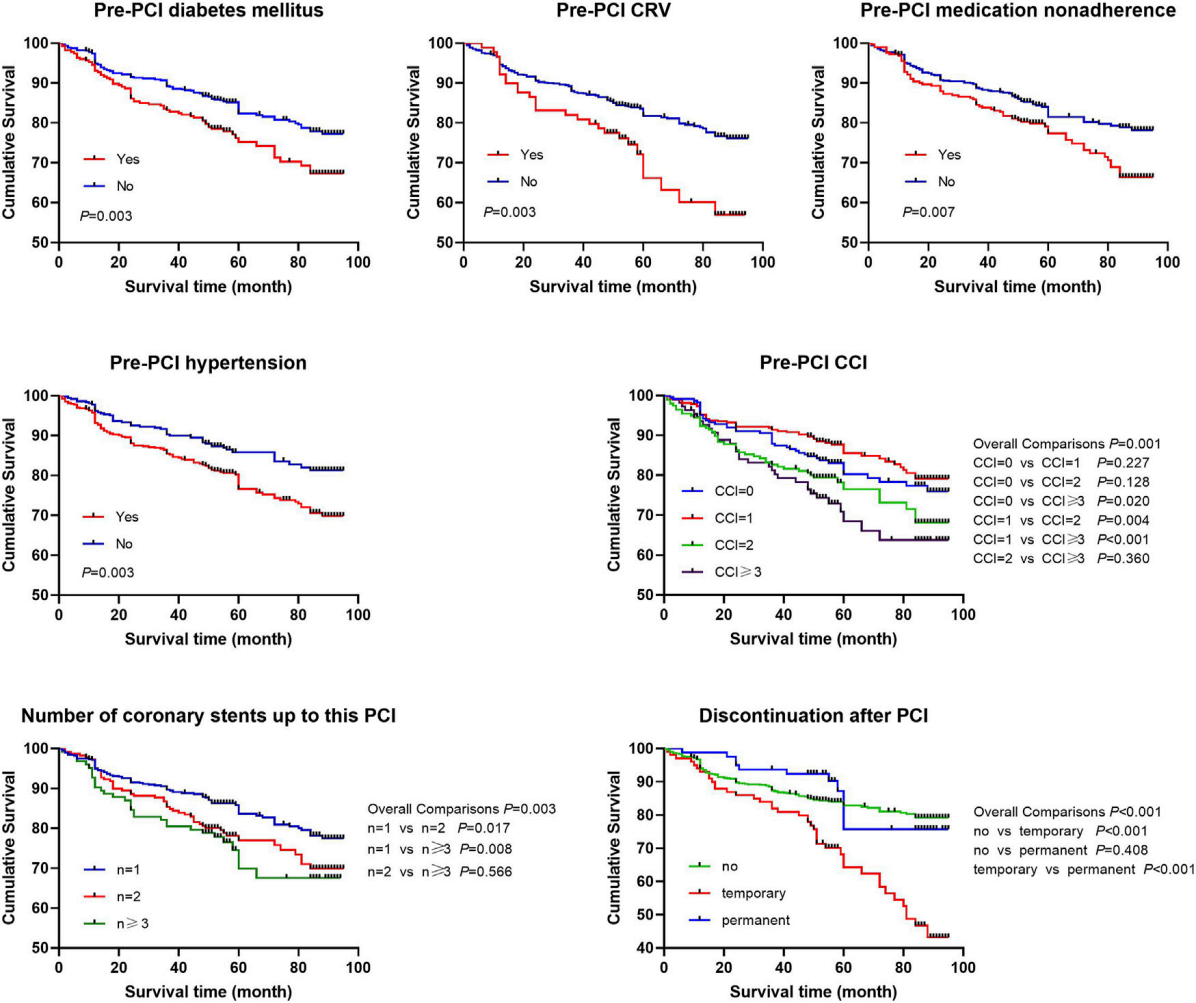
The survival curves after PCI.

The log-rank tests of the Kaplan-Meier analysis showed that pre-PCI CCI, pre-PCI hypertension, pre-PCI diabetes mellitus, pre-PCI CRV, pre-PCI medication nonadherence, number of coronary stents up to this PCI, and discontinuation after PCI were associated with MACE after PCI in the survival curves (Figure 3). Discontinuation after PCI did not meet the proportional hazards hypothesis, and the survival curves of no discontinuation and permanent discontinuation crossed at 60 months.

Univariate analysis showed that (Table 2) place of residence, province, lack of medication necessity, economic difficulties, side effects, complex prescriptions, inconvenience, and routine disruption were associated with permanent discontinuation; place of residence, pre-PCI medication nonadherence and all barriers to medication adherence except extensive travel were associated with temporary discontinuation; pre-PCI CCI, pre-PCI hypertension, pre-PCI diabetes mellitus, pre-PCI CRV, pre-PCI medication nonadherence, number of coronary stents up to this PCI, time after PCI, and discontinuation were associated with MACE. These results were consistent with the unadjusted estimates from univariate Cox regression (Table 3). All of the above potential variables met the proportional hazards hypothesis except discontinuation after PCI.

**TABLE 2:** Univariate associations with discontinuation and MACE after PCI.

Variables, N (%)	Permanent discontinuation			Temporary discontinuation			MACE		
	Yes (N = 80)	No (N = 821)	*p-*value	Yes (N = 100)	No (N = 801)	*p-*value	Yes (N = 180)	No (N = 721)	*p-*value
*Demographics*									
Male	60 (75.0)	569 (69.3)	0.290	71 (71.0)	558 (69.7)	0.784	124 (68.9)	505 (70.0)	0.763
Age, years			0.838			0.472			0.410
<50	7 (8.8)	99 (12.1)		15 (15.0)	91 (11.4)		17 (9.4)	89 (12.3)	
50–59	19 (23.8)	183 (22.3)		21 (21.0)	181 (22.6)		36 (20.0)	166 (23.0)	
60–69	30 (37.5)	309 (37.6)		41 (41.0)	298 (37.2)		76 (42.2)	263 (36.5)	
≥70	24 (30.0)	230 (28.0)		23 (23.0)	231 (28.8)		51 (28.3)	203 (28.2)	
Place of residence			<0.001			0.001			0.239
Large city	7 (8.8)	363 (44.2)		30 (30.0)	340 (42.4)		64 (35.6)	306 (42.4)	
Small to medium-sized city	28 (35.0)	219 (26.7)		22 (22.0)	225 (28.1)		53 (29.4)	194 (26.9)	
Rural area	45 (56.3)	239 (29.1)		48 (48.0)	236 (29.5)		63 (35.0)	221 (30.7)	
Province			0.003			0.123			0.911
Chongqing	52 (65.0)	651 (79.3)		72 (72.0)	631 (78.8)		141 (78.3)	562 (77.9)	
Another province	28 (35.0)	170 (20.7)		28 (28.0)	170 (21.2)		39 (21.7)	159 (22.1)	
*Pre-PCI clinical characteristics*									
CCI			0.253			0.089			0.006
CCI = 0	27 (33.8)	197 (24.0)		25 (25.0)	199 (24.8)		45 (25.0)	179 (24.8)	
CCI = 1	29 (36.3)	342 (41.7)		43 (43.0)	328 (40.9)		57 (31.7)	314 (43.6)	
CCI = 2	14 (17.5)	183 (22.3)		14 (14.0)	183 (22.8)		46 (25.6)	151 (20.9)	
CCI≥3	10 (12.5)	99 (12.1)		18 (18.0)	91 (11.4)		32 (17.8)	77 (10.7)	
Smoking	49 (61.3)	460 (56.0)	0.369	57 (57.0)	452 (56.4)	0.914	102 (56.7)	407 (56.4)	0.958
Alcohol consumption	18 (22.5)	163 (19.9)	0.573	18 (18.0)	163 (20.3)	0.580	28 (15.6)	153 (21.2)	0.090
Hypertension	45 (56.3)	494 (60.2)	0.495	61 (61.0)	478 (59.7)	0.799	124 (68.9)	415 (57.6)	0.006
Hyperlipidemia	26 (32.5)	278 (33.9)	0.806	31 (31.0)	273 (34.1)	0.539	61 (33.9)	243 (33.7)	0.962
Diabetes mellitus	23 (28.7)	252 (30.7)	0.718	31 (31.0)	244 (30.5)	0.912	69 (38.3)	206 (28.6)	0.011
Myocardial infarction	25 (31.3)	232 (28.3)	0.572	27 (27.0)	230 (28.7)	0.720	59 (32.8)	198 (27.5)	0.158
Stroke	9 (11.3)	90 (11.0)	0.937	7 (7.0)	92 (11.5)	0.176	25 (13.9)	74 (10.3)	0.164
CRV	9 (11.3)	80 (9.7)	0.667	5 (5.0)	84 (10.5)	0.083	26 (14.4)	63 (8.7)	0.022
Medication nonadherence	30 (37.5)	262 (31.9)	0.308	45 (45.0)	247 (30.8)	0.004	74 (41.1)	218 (30.2)	0.005
*Vascular conditions up to this PCI*									
Number of coronary stents			0.163			0.119			0.031
1	57 (71.3)	499 (60.8)		70 (70.0)	486 (60.7)		96 (53.3)	460 (63.8)	
2	16 (20.0)	205 (25.0)		22 (22.0)	199 (24.8)		52 (28.9)	169 (23.4)	
≥3	7 (8.8)	117 (14.3)		8 (8.0)	116 (14.5)		32 (17.8)	92 (12.8)	
Noncoronary Atherosclerosis/stenosis	19 (23.8)	263 (32.0)	0.132	36 (36.0)	246 (30.7)	0.282	56 (31.1)	226 (31.3)	0.952
Noncoronary stents	1 (1.3)	18 (2.2)	1.000	1 (1.0)	18 (2.2)	0.712	3 (1.7)	16 (2.2)	0.779
*Post-PCI variables*									
Time, years			0.172			0.110			0.001
4	54 (67.5)	490 (59.7)		53 (53.0)	491 (61.3)		89 (49.4)	455 (63.1)	
7	26 (32.5)	331 (40.3)		47 (47.0)	310 (38.7)		91 (50.6)	266 (36.9)	
Barriers to medication adherence									
Lack of medication necessity	54 (67.5)	69 (8.4)	<0.001	37 (37.0)	86 (10.7)	<0.001	26 (14.4)	97 (13.5)	0.729
Economic difficulties	21 (26.3)	54 (6.6)	<0.001	24 (24.0)	51 (6.4)	<0.001	17 (9.4)	58 (8.0)	0.543
Memory decline	4 (5.0)	53 (6.5)	0.610	12 (12.0)	45 (5.6)	0.013	10 (5.6)	47 (6.5)	0.635
Side effects	19 (23.8)	30 (3.7)	<0.001	11 (11.0)	38 (4.7)	0.009	10 (5.6)	39 (5.4)	0.938
Complex prescriptions	10 (12.5)	28 (3.4)	0.001	14 (14.0)	24 (3.0)	<0.001	5 (2.8)	33 (4.6)	0.283
Inconvenience	13 (16.3)	24 (2.9)	<0.001	9 (9.0)	28 (3.5)	0.016	12 (6.7)	25 (3.5)	0.053
Disturbing routine	9 (11.3)	24 (2.9)	0.001	14 (14.0)	19 (2.4)	<0.001	7 (3.9)	26 (3.6)	0.857
Emotional distress	4 (5.0)	28 (3.4)	0.519	15 (15.0)	17 (2.1)	<0.001	7 (3.9)	25 (3.5)	0.785
Busy	5 (6.3)	23 (2.8)	0.094	9 (9.0)	19 (2.4)	0.002	7 (3.9)	21 (2.9)	0.500
Extensive travel	4 (5.0)	20 (2.4)	0.157	5 (5.0)	19 (2.4)	0.174	4 (2.2)	20 (2.8)	0.801
Discontinuation after PCI									<0.001
None discontinuation							128 (71.1)	593 (82.2)	
Permanent discontinuation							10 (5.6)	70 (9.7)	
Temporary discontinuation							42 (23.3)	58 (8.0)	

**TABLE 3:** Factors associated with discontinuation and MACE after PCI.

Variables	Unadjusted estimates		Adjusted estimates	
	*HR (95%CI)*	*p-value*	*HR (95%CI)*	*p-value*
Factors associated with temporary discontinuation				
Place of residence				
Large city	References			
Small to medium-sized city	1.10 (0.63, 1.90)	0.745		
Rural area	2.22 (1.41, 3.50)	0.001		
Pre-PCI medication nonadherence	1.75 (1.18, 2.60)	0.005	1.63 (1.09, 2.43)	0.016
Lack of medication necessity	4.13 (2.75, 6.20)	<0.001	2.33 (1.44, 3.78)	0.003
Economic difficulties	3.79 (2.39, 6.00)	<0.001	2.09 (1.26, 3.47)	0.005
Memory decline	2.00 (1.10, 3.67)	0.024		
Side effects	2.19 (1.17, 4.10)	0.014		
Complex prescriptions	4.31 (2.45, 7.60)	<0.001		
Inconvenience	2.48 (1.25, 4.92)	0.009		
Disturbing routine	5.22 (2.96, 9.20)	<0.001	2.09 (1.10, 3.99)	0.025
Emotional distress	6.11 (3.52, 10.59)	<0.001	2.76 (1.50, 5.09)	0.001
Busy	3.42 (1.72, 6.78)	<0.001		
Factors associated with permanent discontinuation				
Place of residence				0.002
Large city	References		References	
Small to medium-sized city	6.30 (2.75, 14.42)	<0.001	4.21 (1.82, 9.73)	0.001
Rural area	8.967 (4.04, 19.89)	<0.001	4.18 (1.84, 9.46)	0.001
Province	1.94 (1.22, 3.07)	0.007		
Lack of medication necessity	16.11 (10.08, 25.75)	<0.001	10.60 (6.45, 17.41)	<0.001
Economic difficulties	4.41 (2.68, 7.26)	<0.001		
Side effects	6.41 (3.83, 10.74)	<0.001	3.30 (1.94, 5.62)	<0.001
Complex prescriptions	3.46 (1.78, 6.72)	<0.001		
Inconvenience	5.03 (2.77, 9.11)	<0.001		
Disturbing routine	3.69 (1.84, 7.38)	<0.001		
Factors associated with MACE				
Pre-PCI CCI				
CCI = 0	References		References	
CCI = 1	0.78 (0.53, 1.15)	0.210	0.74 (0.50, 1.09)	0.127
CCI = 2	1.32 (0.88, 2.00)	0.181	1.36 (0.89, 2.06)	0.152
CCI≥3	1.67 (1.06, 2.63)	0.026	1.45 (0.92, 2.30)	0.114
Pre-PCI hypertension	1.54 (1.12, 2.11)	0.008	1.42 (1.04, 1.96)	0.030
Pre-PCI diabetes mellitus	1.53 (1.13, 2.06)	0.006		
Pre-PCI CRV	1.66 (1.10, 2.52)	0.017		
Pre-PCI medication nonadherence	1.49 (1.11, 2.00)	0.009		
Number of coronary stents up to this PCI				
1	References		References	
2	1.44 (1.03, 2.02)	0.035	1.42 (1.01, 1.99)	0.045
≥3	1.67 (1.12, 2.50)	0.012	1.66 (1.11, 2.49)	0.014
Discontinuation after PCI				
None discontinuation	References		References	
Permanent discontinuation				
≤60 months	0.74 (0.38, 1.41)	0.354	0.79 (0.41, 1.50)	0.468
>60 months	<0.001 (0, Inf)	0.991	<0.001 (0, Inf)	0.992
Temporary discontinuation				
≤60 months	2.01 (1.36, 2.97)	<0.001	2.18 (1.47, 3.25)	<0.001
>60 months	7.88 (3.27, 18.95)	<0.001	8.82 (3.65, 21.28)	<0.001

Multivariate Cox regression analysis with adjusted hazard ratio (HR) showed that (Table 3), pre-PCI medication nonadherence (HR 1.63; 95% CI: 1.09–2.43), lack of medication necessity (HR 2.33; 95% CI: 1.44–3.78), economic difficulties (HR 2.09; 95% CI: 1.26–3.47), routine disruption (HR 2.09; 95% CI: 1.10–3.99), and emotional distress (HR 2.76; 95% CI: 1.50–5.09) were associated with temporary discontinuation after PCI; compared with living in a large city, living in a rural area (HR 4.18; 95% CI: 1.84–9.46) or small to medium-sized city (HR 4.21; 95% CI: 1.82–9.73), lack of medication necessity (HR 10.60; 95% CI: 6.45–17.41) and side effects (HR 3.30; 95% CI: 1.94–5.62) were associated with permanent discontinuation after PCI. For discontinuation, according to the point of crossed survival curves, the time was divided into two periods, namely, ≤60 months and >60 months, and its association with MACE was analyzed separately. The multivariate time-dependent Cox regression showed that pre-PCI hypertension (HR 1.42; 95% CI: 1.04–1.96), two coronary stents (HR 1.42; 95% CI: 1.01–1.99) or three coronary stents (HR 1.66; 95% CI: 1.11–2.49) compared to one coronary stent up to this PCI, and temporary discontinuation (≤60 months HR 2.18; 95% CI: 1.47–3.25; >60 months HR 8.82; 95% CI: 3.65–21.28) were associated with MACE. It is evident that temporary discontinuation and permanent discontinuation were associated with different factors, and temporary discontinuation was associated with MACE after PCI.

## Discussion

We found that discontinuation and MACE after PCI had similar temporal characteristics, both with the highest incidence in the first year after PCI, and then either rapidly or slowly declining. Pre-PCI CCI, pre-PCI hypertension, pre-PCI diabetes mellitus, pre-PCI CRV, pre-PCI medication nonadherence, number of coronary stents up to current PCI, and temporary discontinuation after PCI were associated with the survival curves. The factors associated with temporary discontinuation and permanent discontinuation are different. Pre-PCI medication nonadherence, lack of medication necessity, economic difficulties, routine disruption, and emotional distress were associated with temporary discontinuation, whereas place of residence, lack of medication necessity, and side effects were associated with permanent discontinuation. Pre-PCI hypertension, number of coronary stents up to this PCI, and temporary discontinuation after PCI were associated with MACE after PCI. These findings may provide important guidance for medication adherence interventions in patients undergoing PCI.

The temporal characteristics of discontinuation and MACE after PCI had a similar pattern, which may reflect the same reason. Because temporary discontinuation was associated with MACE in the survival curves and time-dependent Cox regression analyses, this temporal characteristic may reflect the impact of medication adherence on adverse outcomes (Kronish et al., 2020; Princip et al., 2023). However, the existing literature also shows that other outcomes and psychological symptoms after PCI have a similar temporal characteristic (Gu et al., 2016; Williams et al., 2021; Murad et al., 2022; Vulcanescu et al., 2023). Therefore, in addition to the effect of medication adherence on outcomes, this temporal characteristic may also reflect the physical and psychological stress effect of ischemic heart disease or PCI (Vilchinsky et al., 2017; Birk et al., 2019; Kronish et al., 2020; Henein et al., 2022; Princip et al., 2023). The stress effect diminishes over time, resulting in a trend toward a decrease in associated medication nonadherence, psychological symptoms, and clinical outcomes. Thus, medication nonadherence after PCI may also be a consequence of patient maladaptation to the stress of the disease and/or PCI (Vilchinsky et al., 2017; Kronish et al., 2020; Narisawa et al., 2021; Princip et al., 2023). Clinical outcomes, on the other hand, may be influenced by both medication nonadherence and the stress of disease and/or PCI (Vilchinsky et al., 2017; Henein et al., 2022; Princip et al., 2023). Future work is needed to test these possibilities and to explore the possibility of developing medication adherence interventions from a stress perspective for patients undergoing PCI (Birk et al., 2019; von Känel et al., 2022).

Differences in the factors associated with temporary and permanent discontinuation may have interesting implications for medication adherence interventions. This difference may be relative and due to the fact that temporary and permanent discontinuation belong to different phases of medication adherence, namely, the implementation and persistence. Importantly, these differences may suggest that the two patterns of discontinuation require different models of medication adherence interventions. The lack of medication necessity is associated with both types of medication discontinuation, but it may be more closely associated with permanent discontinuation, in terms of its HR values. It can be concluded that discontinuations related to pre-PCI medication nonadherence, financial difficulties, routine disruption, and emotional distress are more likely to be transient and less difficult to intervene with, and tailored interventions may be appropriate (Xu et al., 2020). In contrast, discontinuation due to lack of medication necessity (West et al., 2020), living in a nonmetropolitan area (Pietrzykowski et al., 2020; Simon et al., 2021), and side effects (Zhou et al., 2021; Golder et al., 2022; Redfors et al., 2022; Yamamoto et al., 2023) may be more likely to be permanent and more difficult to intervene with, and an integrative consideration of patient perceptions, healthcare resources, medication characteristics, and therapeutic guidelines is needed to maximize benefit (Simon et al., 2021).

We found that temporary discontinuation was associated with MACE in survival curves and time-dependent Cox regression results, whereas permanent discontinuation was not. This suggests that temporary discontinuation after PCI may have a significant effect on MACE (Sorrentino et al., 2020; Redfors et al., 2022; Yamamoto et al., 2023), which is the basis for the need for adherence interventions. On the other hand, it suggests that permanent discontinuation after PCI has a nonsignificant effect on MACE, which is inconsistent with the results of previous studies and may be related to the design of our telephone survey (Xie et al., 2019). In clinical practice, certain medications, such as P2Y12 inhibitors and β-blockers, can be discontinued under physician supervision at specific times after PCI in certain patients without being associated with an increased risk of MACE (Sorrentino et al., 2020; Redfors et al., 2022; Halili et al., 2023; Kinlay et al., 2023; Park et al., 2023). Our telephone survey did not differentiate between the types of medications discontinued by self-administration, which may have led patients to confuse self-discontinuation with physician-guided discontinuation and to categorize physician-guided discontinuation as self-discontinuation. It is necessary to re-validate the current results again by distinguishing between medication classes in the future.

Our study provides guidance for medication adherence interventions after PCI. First, we found that both discontinuation and MACE had the highest incidence in the first year after PCI and then either rapidly or slowly decreased. We hypothesized that the stress of disease and/or PCI may be the common factors influencing discontinuation and outcomes after PCI. Therefore, theoretical constructs and practical tests of medication adherence interventions from a stress perspective would be worth trying. Second, we found that temporary and permanent discontinuation were associated with two different types of factors, which may require different intervention patterns. Third, temporary discontinuation after PCI associated with MACE provided evidence for the need for medication adherence interventions. Taken together, our study strengthens the rationale for medication adherence interventions in patients undergoing PCI and informs their theoretical construction and practical implementation.

Our study has shortcomings. First, we used a combination of patient self-report and medical records to assess discontinuation without differentiating medication classes for ease of use. Patient self-report may overestimate medication adherence (Fanaroff et al., 2020; Lauffenburger et al., 2020). More importantly, failure to categorize medications may confuse patients, as we discussed above, as well as adversely affect the association of discontinuation of different medications with MACE. These shortcomings may limit the generalizability of our research findings, as well as be disadvantageous for tailored medication adherence intervention. Therefore, it is necessary to combine objective assessments of medication adherence and categorize medication classes in the future (Kronish et al., 2021). Second, the medication adherence process includes multiple indicators other than discontinuation (Ho et al., 2009; Vrijens et al., 2012), and discontinuation in this study is only one of them, and the scope of investigation can be comprehensive enough to include more indicators in the future. Third, we discussed the possible psychological mechanisms of patients undergoing PCI related to our findings, which is mainly a theoretical speculation based on the existing literature and psychological general knowledge, which needs to be investigated by clinical trials.

## Conclusions

To provide guidance for interventions to improve medication adherence after PCI, we analyzed the temporal characteristics and associated factors of discontinuation and MACE after PCI in patients with ischemic heart disease. Discontinuation and MACE had similar temporal characteristics, which may reflect the stressful effects of disease and/or PCI on medication adherence and outcomes. The factors associated with temporary and permanent discontinuation are different, which may suggest that they require different intervention patterns. Temporary discontinuation was associated with MACE after PCI, highlighting the need for medication adherence interventions. Permanent discontinuation was not associated with MACE after PCI, which may be related to the design of the telephone survey and needs to be retested in the future with differentiation of medications.

## Data Availability

The original contributions presented in the study are included in the article/Supplementary Material, further inquiries can be directed to the corresponding author.
