# Supplementary material for: Temporal characteristics and associated factors of discontinuation and outcomes after percutaneous coronary intervention
Source: Front Pharmacol. 2024 Apr 9;15:1355231. doi: 10.3389/fphar.2024.1355231 (PMC11035793; doi:10.3389/fphar.2024.1355231)
Supplement: Supplementary file 1 [file Table1.docx]

**Supplement**

**Table 1 Annual Distribution of Discontinuation and Outcomes after PCI**

|  | Year after PCI | | | | | | | |
| --- | --- | --- | --- | --- | --- | --- | --- | --- |
|  | 1st | 2nd | 3rd | 4th | 5th | 6th | 7th | 8th |
| Temporary discontinuation | 45 | 20 | 8 | 18 | 1 | 3 | 5 | 0 |
| Permanent discontinuation | 37 | 17 | 16 | 7 | 3 | 0 | 0 | 0 |
| Sum of discontinuation | 82 | 37 | 24 | 25 | 4 | 3 | 5 | 0 |
| Cardiac death | 5 | 1 | 5 | 3 | 1 | 4 | 2 | 0 |
| Myocardial infarction | 5 | 4 | 1 | 1 | 5 | 1 | 5 | 0 |
| Stroke | 11 | 8 | 5 | 12 | 6 | 4 | 1 | 1 |
| CRV | 35 | 33 | 12 | 12 | 15 | 3 | 9 | 2 |
| Sum of cardiac death, myocardial infarction and stroke | 21 | 13 | 11 | 16 | 12 | 9 | 8 | 1 |
| Sum of MACE | 56 | 46 | 23 | 28 | 27 | 12 | 17 | 3 |
